# PKCα-induced drug resistance in pancreatic cancer cells is associated with transforming growth factor-β1

**DOI:** 10.1186/1756-9966-29-104

**Published:** 2010-08-05

**Authors:** Ying Chen, Guanzhen Yu, Danghui Yu, Minghua Zhu

**Affiliations:** 1 Department of Pathology, Changhai Hospital, Second Military Medical University, Shanghai, 200433, China; 2 Department of Oncology, Changzheng Hospital, Second Military Medical University, Shanghai, 200433, China

## Abstract

**Background:**

Drug resistance remains a great challenge in the treatment of pancreatic cancer. The goal of this study was to determine whether TGF-β1 is associated with drug resistance in pancreatic cancer.

**Methods:**

Pancreatic cancer BxPC3 cells were stably transfected with TGF-β1 cDNA. Cellular morphology and cell cycle were determined and the suppressive subtracted hybridization (SSH) assay was performed to identify differentially expressed genes induced by TGF-β1. Western blotting and immunohistochemistry were used to detect expression of TGF-β1-related genes in the cells and tissue samples. After that, the cells were further treated with an anti-cancer drug (e.g., cisplatin) after pre-incubated with the recombinant TGF-β1 plus PKCα inhibitor Gö6976. TGF-β1 type II receptor, TβRII was also knocked down using TβRII siRNA to assess the effects of these drugs in the cells. Cell viability was assessed by MTT assay.

**Results:**

Overexpression of TGF-β1 leads to a markedly increased invasion potential but a reduced growth rate in BxPC3 cells. Recombinant TGF-β1 protein increases expression of PKCα in BxPC3 cells, a result that we confirmed by SSH. Moreover, TGF-β1 reduced the sensitivity of BxPC3 cells to cisplatin treatment, and this was mediated by upregulation of PKCα. However, blockage of PKCα with Gö6976 and TβRII with siRNA reversed the resistance of BxPC3 cells to gemcitabine, even in the presence of TGF-β1. Immunohistochemical data show that pancreatic cancers overexpress TGF-β1 and P-gp relative to normal tissues. In addition, TGF-β1 expression is associated with P-gp and membranous PKCα expression in pancreatic cancer.

**Conclusions:**

TGF-β1-induced drug resistance in pancreatic cancer cells was associated with PKCα expression. The PKCα inhibitor Gö6976 could be a promising agent to sensitize pancreatic cancer cells to chemotherapy.

## Background

Drug resistance poses a significant challenge to achieving clinical control of pancreatic cancer. Resistance to chemotherapy frequently results in disease relapse and tumor recurrence, leading to shorter survival times for patients with pancreatic cancer than those with other gastrointestinal cancers. Elimination or minimization of drug resistance will improve our ability to control pancreatic cancer and increase patient survival. However, there are multiple etiologies for drug resistance, and they are not well understood.

PKCα is a classic member of the protein kinase C family, and some studies have demonstrated an association between PKCα and drug resistance in human cancers [1,2]. PKCα-associated drug resistance is likely mediated by P-gp, which is encoded by the multidrug resistant gene 1 (*MDR1*) gene. P-gp belongs to the ATP-binding cassette (ABC) transporter superfamily, and it functions as a drug efflux pump in multidrug resistance. PKCα modulates the function of P-gp via phosphorylation of the P-gp intracellular domain or activation of the *MDR1 *gene promoter. Curcumin [3], hammerhead ribozymes [4], and antisense oligonucleotides [5], which all target P-gp, have been shown to improve the efficacy of chemotherapy in a variety of cancer models. However, the molecular mechanism of PKCα/P-gp-initiated drug resistance in pancreatic cancer is poorly understood.

There are three subtypes of transforming growth factor-β in humans: TGF-β1, TGF-β2, and TGF-β3. This growth factor is upregulated in some human cancers, and the various subtypes play crucial roles in tissue regeneration, cell differentiation, embryonic development, and regulation of the immune system. TGF-β1 is a multifunctional cytokine endowed with both anti-neoplastic and pro-oncogenic activities in human cancers. TGF-β1 has been shown to enhance the efficacy of anti-cancer drugs by repressing cellular proliferation [6-10]. Smad4 mediates the anti-neoplastic activities of TGF-β1 (such as inhibition of tumor cell growth and induction of apoptosis [11-14]. For example, TGF-β1 induces the antitumor activity of dihydrotestosterone (DTH) in prostate cancer by causing the tumor cells to undergo apoptosis. This effect is mediated through Smad4, which negatively regulates the growth of epithelial cells and the extracellular matrix (ECM) [15].

SMAD4 is mutated in many cancers, including pancreatic cancer. It is a tumor suppressor gene that regulates the TGF-β signal transduction pathway. Indeed, several studies have demonstrated that TGF-β1 promotes invasiveness and metastasis if Smad4 is absent or mutated via a Smad4-independent pathway [16-19]. To date, no one has reported a correlation between TGF-β1 and chemotherapy resistance in pancreatic cancer.

The information presented above suggests that Smad4-dependent and -independent signaling pathways regulate cancer cell resistance to chemotherapy. This is particularly important in pancreatic cancer chemotherapy because more than 50% of pancreatic cancers have inactivated Smad4 protein [20], which may result in activation of the Smad4-independent TGF-β1 pathway when patients undergo such treatment. In this study, we determined whether TGF-β1 is associated with drug resistance in pancreatic cancer and then explored the possible underlying mechanism. TGF-β1 induces drug resistance in a Smad4-null pancreatic cancer cell line. The effect of TGF-β1 was mediated by PKCα/P-gp and the epithelial-to-mesenchymal transition (EMT). Moreover, a selective inhibitor of PKCα, Gő6976, was able to reverse the effects of TGF-β1-induced drug resistance in pancreatic cancer cells.

## Materials and methods

### Cell line and tissue samples

The human pancreatic cancer cell line BxPC3, which shows homogeneous loss of SMAD4, was generously provided by Dr. Zhao-shen Li of the Department of Gastroenterology, Changhai Hospital, Shanghai. The cells were grown in Dulbecco's modified Eagle's medium (DMEM) plus 10% fetal bovine serum, 100 U/ml of penicillin and streptomycin (all were from Invitrogen-Gibco, Carlsbad, CA, USA) at 37°C in a humidified atmosphere of 95% air and 5% CO_2_. Tissue specimens from 42 pancreatic ductal adenocarcinoma patients were obtained from the Department of Pathology at Changhai Hospital, which is affiliated with the Second Military Medical University, Shanghai, China. Our institutional review board approved the use of tissue samples, and the patients all provided informed consent.

### Reagents

Human recombinant TGF-β1 was purchased from Peprotech (Rocky Hill, NJ, USA) and dissolved in phosphate-buffered saline (PBS) (pH 7.2) with 1 mg/ml bovine serum albumin (BSA, from Amresco, USA). Gö6976, a selective inhibitor of PKCα, was purchased from Biosource (San Jose, CA, USA) and used at concentrations of 100 nM, 1 M and 10 M. Anti-cancer drugs (5-FU, gemcitabine, oxaliplatin, cisplatin, CPT-11 and epirubicin) were obtained from the Department of Oncology of Changzheng Hospital, Shanghai, China.

### Gene transfection, cellular morphological changes and mobility assay

A pcDNA3 vector containing full-length cDNA for TGF-β1 was obtained from the Department of Pathology, Fudan University, China. BxPC3 cells were transfected with the pcDNA3/TGF-β1 plasmid or pcDNA3 as a mock control using the Lipofectamine™ 2000 transfection kit (Invitrogen). The cells were then fed with fresh selective medium containing 800 μg/ML G418 (Invitrogen-Gibco) for 2-3 weeks, and stable gene-transfected cell clones were individually transferred into six-well plates for expansion to establish sublines that stably expressed the gene product. TGF-β1 expression was confirmed by Western blot analysis. Cellular morphology was observed using an inverted phase contrast microscope (x40) and photographed with a digital camera (Olympus, Japan).

For the wound healing assay, cells were plated in 24-well cell culture plates. After they reached confluence, a plastic pipette tip was drawn across the center of the plates to produce a clean 1 millimeter-wide wound area. Cell migration into the wound area was examined 24 hours after culturing in DMEM with 10% FBS.

### Protein extraction and western blotting

Cells were grown in DMEM for 3 days, and total cellular proteins were isolated using a cell lysis buffer containing phosphatase inhibitor (Merck, Germany). The protein concentration was then measured with a BioRad Protein Assay Kit II (BioRad Laboratories, Hercules, CA) according to the manufacturer's protocol. Samples containing 50 μg of protein from the cells were separated by 10-14% polyacylamide SDS-PAGE gels and then transferred electrophoretically to a Hybond-C nitrocellulose membrane (GE Healthcare, Arlington Heights, IL) at 500 mA for 2 h at 4°C. The membrane was subsequently stained with 0.5% Ponceau S containing 1% acetic acid to confirm that the proteins were loaded equally and to verify transfer efficiency. The membranes were next incubated overnight in a blocking solution containing 5% bovine skim milk and 0.1% Tween 20 in PBS at 4°C. The next day, the membranes were incubated with primary antibodies for 2 h at room temperature. The antibodies used were anti-TGF-β1 polyclonal antibody (sc-146), anti-p21*^WAF1 ^*monoclonal antibody (sc-817), anti-cyclinD1 polyclonal antibody (sc-20044), anti-SMA monoclonal antibody (sc-56499), anti-GAPDH polyclonal antibody (sc-20357) (all from Santa Cruz Biotechnology, Inc., Santa Cruz, CA, USA), anti-p38 polyclonal antibody (B205405) (Stressgen, Ann Arbor, MI, USA), anti-p-p38 polyclonal antibody (AF1576), anti-ERK1/2 polyclonal antibody (AF1576), anti-pERK1/2 polyclonal antibody (AF1018) (R&D Systems, Minneapolis, MN, USA), anti-P-gp (MS-660) (Lab Vision, Fremont, CA, USA,) anti-TβRII polyclonal antibody (cst-2518) and anti-PKCα polyclonal antibody (cst-2056) (Cell Signal Technology, Beverly, MA, USA). The membranes were washed in PBS and incubated for 1.5 h with a chemiluminescent system for HRP-conjugated antibodies (Santa Cruz Biotechnology) to visualize the protein bands on X-ray film.

### Immunohistochemical analysis

Tissue sections (4-μm) were cut from paraffin blocks and deparaffinized by routine procedures. Immunohistochemical analyses were performed by using the DAKO system (DOKO, Carpinteria, CA, USA), and DAB was used as the chromogen. The tissue sections were counterstained with hematoxylin. The primary antibodies used included monoclonal anti-PKCα antibody (sc-8393), polyclonal anti-TGF-β1 antibody (sc-146) (Santa Cruz Biotechnology, Inc.) and monoclonal anti-P-gp antibody (M-660-P, from Labvision). The stained sections were reviewed and scored using an Olympus microscope. The sections were then scored as positive or negative according to their staining intensity and percentage of the staining.

### Suppressive subtracted hybridization (SSH) screening

We performed SSH to identify changes in gene expression between stably TGFβ1- and vector-only-transfected BxPC3 cells. Total RNA was isolated from these sublines by using an RNAeasy Mini kit (Qiagen, Santa Clara, CA). Next, total RNA was reversely transcribed into cDNA using a cDNA subtraction kit (Clontech, Mountain View, CA, USA). An excess of driver double-stranded cDNAs, synthesized from poly(A)+RNA, was added to microtubes containing adaptor 1- and adaptor 2-ligand tester cDNA for the first hybridization. After two rounds of hybridization, subtracted or differentially expressed cDNAs were amplified by nested PCR. Products from the secondary PCRs were inserted into the pUCm-T/A cloning vector, and the plasmids were then transformed into the *Escherichia coli *JM109 strain for further screening and identification. The transformants containing subtracted cDNAs were grown on LB agar plates containing 100 μg/ml ampicillin and X-gal (50 μl of a 2 mg/ml stock solution per 100 mm plate), and individual colonies were selected and grown in LB broth at 37°C overnight for identification of differentially expressed genes.

### Dot blotting and DNA sequencing

Reverse Northern blot combined with dot blotting was used to confirm differential expression in the subtracted gene clones. Dots with a higher intensity in the transfected group than those in the mock group were categorized as the upregulation group, and clones with weaker signals were categorized as the downregulation group. All clones with differentially expressed genes were sequenced using a M13 (+) and/or M13 (-) promoter flanking the cloning sites. They were then analyzed with an Applied Biosystems 320 genetic analyzer. Homological searches for gene identification were performed with the online BLASTn and BLASTx programs.

### Cell viability MTT assay

The tetrazolium dye [3-(4,5-Dimethylthiazol-2-yl)-2,5-diphenyltetrazolium bromide, MTT; Sigma-Aldrich, St. Louis, MO, USA] assay was performed to assess cytotoxicity of different chemotherapeutic drugs to the pancreatic cancer cells. Briefly, ten thousand cells were cultivated in 96-well plates with DMEM containing 1% FBS and 5 or 10 ng/ml recombinant TGF-β1 (Peprotech). The controls contained 1% BSA only instead of TGF-β1. To test the effect of Gö6976 in the cancer cells treated with different chemotherapeutic drugs, a range of concentrations of Gö6976 (100 nM, 1 μM, or 10 μM) was added into the culture media together with 5 μg/ml of TGF-β1. After 24 hours, the cells were treated with anti-cancer drugs for an additional 24 hours. Following this incubation, the culture medium was replaced with 100 μl of 0.05% MTT solution, and the cell culture was incubated for 4 hours. The absorption rate was then measured at 490 nm using a microplate reader (Anthos Labtec Instruments, Austria), and the IC_50 _was calculated as the drug concentration that reduced the optical density by 50%.

### Construction of siRNA vector

The pSliencer2.1/U6 vector was purchased from Ambion Company (Austin, TX, USA) to harbor siRNA. We used online tools to design TGF-β typeII receptor-targeting siRNA, and the sequences were 5'-GATCCGTATAACACCAGCAATCCTGTTCAAGAGACAGGATTGCTGGTGTTATATTTTTTGGAAA-3' (sense sequence) and 5'-AGCTTTTCCAAAAAATATAACACCAGCAATCCTGTCTCTTGAACAGGATTGCTGGTGTTATACG-3' (antisense sequence). The DNA oligonucleotides were then synthesized by Invitrogen (Shanghai, China). Next, the sense and antisense DNA oligonucleotides were annealed to form double-stranded DNA, which was inserted into the pSliencer2.1/U6 vector. After the sequences were confirmed and the vector was amplified, this vector was transfected into the pancreatic cancer cell line. After selection with 800 μg/mL of G418 for over three weeks, the sublines were isolated and tested for gene silencing. Once silencing was verified, we used these cells for drug cytotoxicity assays.

### Statistical analyses

Statistical analyses were performed with SPSS 10.0 software. The χ^2 ^test was used to assess immunohistochemical data, and we used an *ANOVA*-test for the MTT assay. All statistical tests were two-sided, and *p *< 0.05 was considered to be statistically significant.

## Results

### Role of TGF-β1 in pancreatic cancer BxPC3 cells

**Figure 1:**
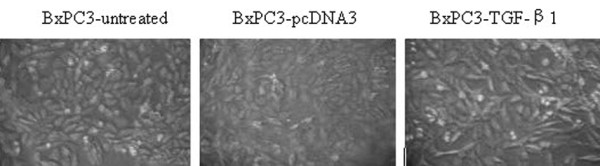
**The effects of TGF-β1 on the cellular morphology in BxPC3 cells**. Cells transfected with TGF-β1 plasmid take on a long spindle shape with less cell-to-cell contact than the untreated group or the mock group. Cells in the latter two groups are oval or blunt shape with close cell contact.

**Figure 2:**
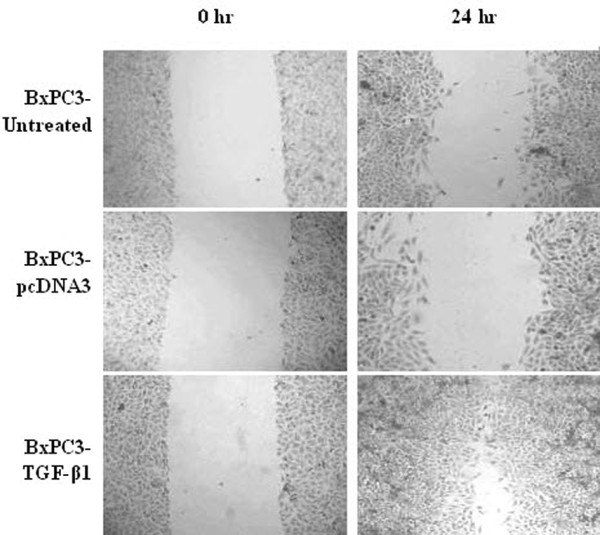
**The effects of TGF-β1 transfection on tumor cell migration**. Pancreatic cancer BxPC3 cells were stably transfected with TGF-β1 and subjected to a migration assay.

**Figure 3:**
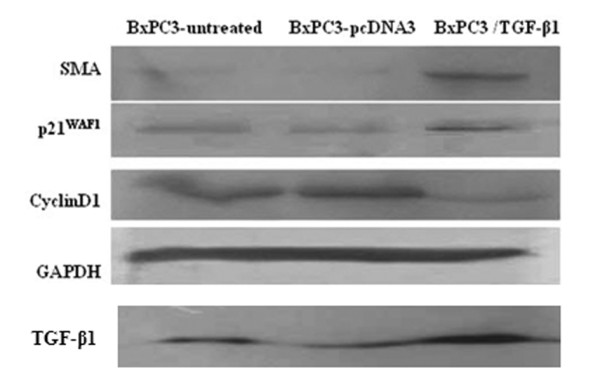
**Western blotting analysis of gene expression**. Stably TGF-β1-transfected BxPC3 cells were grown and treated with G418, and total cellular protein was isolated and subjected to Western blotting analysis. α-SMA, a mesenchymal marker, is responsible for the enhanced cell mobility. CyclinD1 is responsible for cell growth, while p21WAF1 is involved in cell growth arrest.

We stably transfected a TGF-β1 expression vector into BXPC3 cells and then assessed the alterations in phenotype. For example, we first determined the morphological modifications in stably TGF-β1-transfected BxPC3 cells by comparing them to vector-control-transfected sublines. After TGF-β1 transfection, tumor cells underwent obvious morphological changes. In particular, the original oval shape and tightly arranged colonies changed to spindle cells with a stellate shape and less cell-to-cell contact (Figure 1). The wound healing assay shows that BxPC3/TGF-β1 cells recovered from the wound much faster than controls or the parental cell line (Figure 2). Cell cycle analysis by flow cytometry showed a shortened S phase in BxPC3/TGF-β1 cells (17.01 ± 2.65%) compared to parental cells (27.53 ± 2.42%) and cells in the vector-only controls (26.32 ± 1.36%). At the molecular level, expression of α-SMA, a marker of EMT, and p21^WAF1^, an inhibitor of cyclin-dependent kinases, were significantly upregulated, while that of cyclinD1 was reduced in stably TGF-β1-transfected BxPC3 cells (Figure 3).

### TGF-β1 reduced the sensitivity of BxPC3 cells to cisplatin through upregulation of PKCα

**Figure 4:**
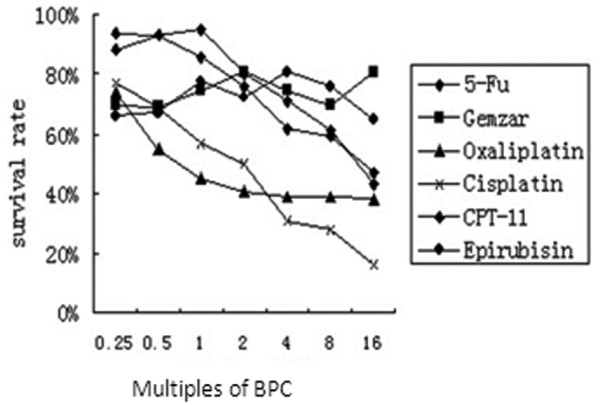
**Resistances of BxPC3 cells to various anti-cancer drugs**. BxPC3 cells were incubated with the drugs for 48 hours. Then cell viability was assayed by MTT. Cisplatin showed the strongest anti-tumor ability, with a typical dose-effect curve; gemcitabine showed almost no effect on cellular survival. BPC, Blood peak concentration of drugs.

**Figure 5:**
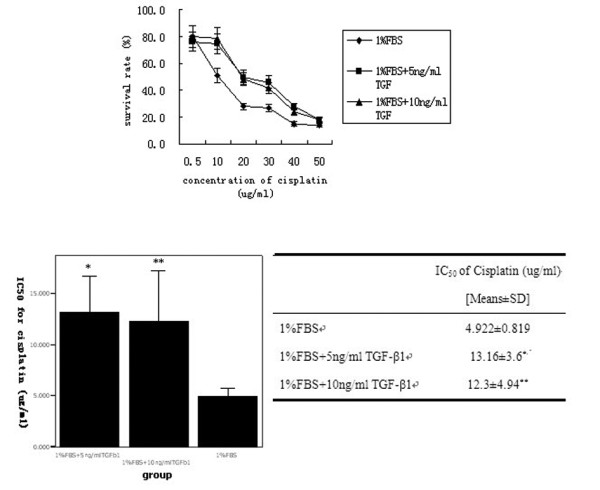
**Effects of TGF-β1 on tumor cell survival**. (A) BxPC3 cells were pre-incubated with 5 and 10 ng/ml of TGF-β1 or 1% FBS as a control for 24 h and then treated with various concentrations of cisplatin for additional 48 h. Cell viability was determined by the MTT assay. (B) IC_50 _values (μg/ml) of cisplatin were calculated based on the above treatment in the tumor cells. *represents significant difference of IC_50 _value between group '1%FBS + 5 ng/ml TGF-β1' and group '1%FBS' (P = 0.03); **represents significant difference between group '1%FBS + 10 ng/ml TGF-β1' and group '1%FBS' (P = 0.044).

**Figure 6:**
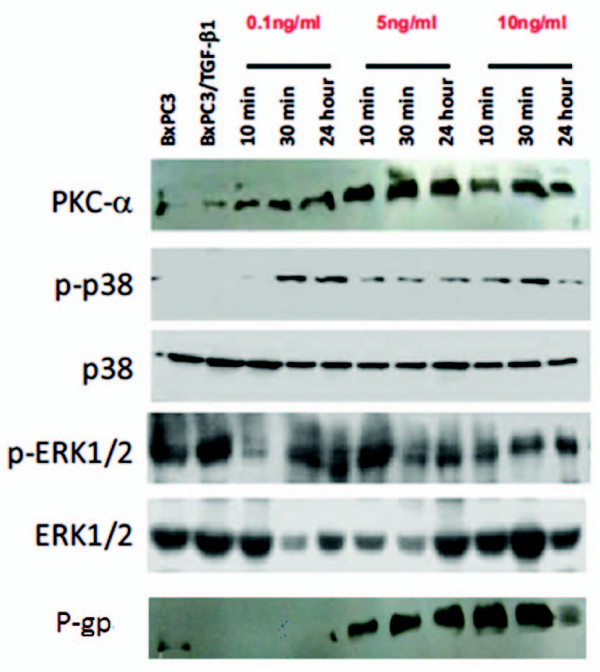
**The effects of TGF-β1 on expression levels of PKCα and p38 MAPK**. BxPC3 cells were treated with 0.1, 1 and 10 ng/ml TGF-β1 for 10 min, 30 min and 24 h. Total cellular protein was extracted and subjected to western blotting analysis to detect expression of PKCα, phosphorylated-p38/total p38 MAPK and phosphorylated-ERK1/2/total ERK1/2. Bx represents BxPC3 cells and Bx/T represents the stably transfected BxPC3 cells with TGF-β1 plasmid.

We first assessed the sensitivity of BXPC3 cells to different chemotherapeutic drugs. The IC_50 _values were 25, 100, 10, 6, 40 and 5 μg/ml for 5-FU, gemcitabine, oxaliplatin, cisplatin, CPT-11, and epirubicin, respectively (Figure 4). We then chose cisplatin for the following experiments. TGF-β1 significantly decreased the sensitivity of BxPC3 cells to cisplatin when the cells were pre-incubated with 5 or 10 ng/ml TGF-β1 before cisplatin treatment (*P *< 0.01, Figure 5). Furthermore, PKCα and P-gp proteins were upregulated in a dose- and time-dependent manner (Figure 6). In addition, TGF-β1 increased p38 phosphorylation, but not ERK1/2 phosphorylation (Figure 6).

**Figure 7:**
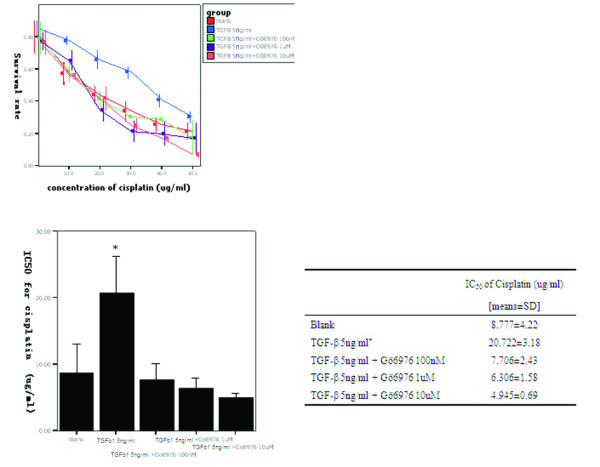
**MTT assay**. (A) BxPC3 cells were grown in DMEM containing 5 μg/ml of TGF-β1 and then treated with or without Gö6976, an inhibitor of PKCα at the indicated concentrations. After this pretreatment, the cells were further treated with cisplatin for an additional 48 h, and the cell viability was determined via MTT assay. (B) IC_50 _values. * represents a significant difference in IC_50 _values between groups for TGF-β1 (5 ng/ml) and all other groups.

To determine whether the induced PKCα activity is responsible for the TGF-β1-induced decrease in the sensitivity of BxPC3 cells to cisplatin, we treated the cells with a selective PKCα inhibitor, Gö6976, and assessed TGF-β1-induced drug resistance. We found that inhibition of PKCα activity could partially reverse TGF-β1-induced drug resistance of BxPC3 cells to cisplatin (Figure 7).

### Blockade of PKCα and TβRII reversed the resistant status of BxPC3 cells

**Figure 8:**
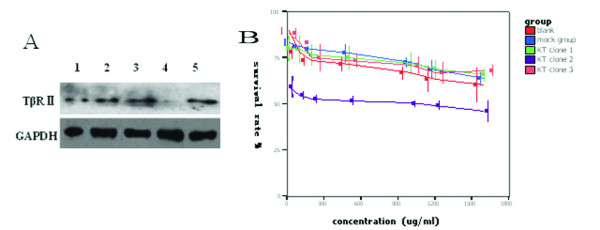
**Role of TβRII siRNA in BxPC3 cells**. (A) Western blotting analysis of TβRII (type II receptor or TGF-β1) protein levels. BxPC3 cells were grown and transfected with TβRII siRNA. After selection with G418, three clones were isolated and the cells from these clones underwent protein isolation. They were subjected to Western blotting analysis with anti-TβRII antibody. Lane 1, total pool of BxPC3 cells; lane 2, mock clone (transfected with empty plasmid, psilenser 2.1 U6); lane 3, knockdown (KD) clone 1; lane 4, KD clone 2; and lane 5, KD clone 3. (B) MTT assay. The transfected BxPC3 cells were grown and treated with gemcitabine at the indicated doses for 2 days. The cell viability was detected by using the MTT assay. The data show that inhibition of TβRII increases sensitivity of BxPC3 cells to gemcitabine. The IC50 value of clone 2 to gemcitabine was the lowest, indicating that clone 2 is more sensitive to gemcitabine than the other cells (*P *< 0.05).

We designed and constructed a TGF-β type II receptor (TβRII) siRNA expression vector to knockdown TβRII expression. We stably transfected the TβRII siRNA vector into BxPC3 cells and isolated three stable clones. Western blotting analysis showed that TβRII expression was significantly knocked down in clone 2 relative to the other two clones (Figure 8A). We chose clone 2 for the following experiments. The IC_50 _of clone 2 to gemcitabine was 812 μg/ml, much lower than that for the vector-only-transfected BxPC3 and the parental cells (Figure 8B), indicating that knockdown of TβRII increases the mortality of cancer cells and increases sensitivity to gemcitabine.

### Detection of differential gene expression after TGF-β1 transfection using an SSH assay

A suppressive subtracted hybridization (SSH) assay was performed to identify differential expression of genes in BXPC3 cells after they were stably transfected with TGF-β1. We found a total of 33 cDNA clones after dot hybridization, out of which 10 genes were upregulated and 13 genes were down-regulated (Table 1). After we BLASTed these clones using online tools, we found that some of the genes are involved in drug resistance (AKR1B10 and PKCα), stromal genesis (MGEA5, FN1, APLP2, PLOD2, WDR1, and CAPZA1), and cell proliferation (eEF1A1, SLC25A3, and SEC61B).

**Table 1:** Differentially expressed genes after stable TGF-β1 transfection

Gene designation	Gene homology	Unigene ID	Gene function	Appearance
**Up-regulated genes**				
EEF1A1	Known	Hs.439552	Protein synthesis	6
PRKCA	Known	Hs.349611	Protein Kinase C-α	2
Homo sapiens chromosome 17, clone RP13-63C9	Unknown		KIAA1554	1
Human DNA sequence from clone RP5-827L5 on chromosome 20	Unknown			1
AKR1B10	Known	Hs.116742	Aldose reductase	1
Homo sapiens 3 BAC RP11-461M2	Unknown			1
FLJ20296	Unknown	Hs.440401	Hypothetical protein	1
MGEA5 (meningioma expressed antigen 5)	Known	Hs.5734	hyaluronidase	1
APLP2	Known	Hs.370247	Amyloid beta 1 precursor-like protein 2	1
FN1	Known	Hs.203717	Fibronectin	1

**Down regulated genes**				
CAPZA1	Known	Hs.309415	Actin filament muscle	1
PLOD2	Known	Hs.41270	Procollagen-lysin	2
PEG10	Unknown	Hs.137476	Predicted protein	1
HNRPDL	Known	Hs.372673	RNA binding protein	3
KIAA1423	Unknown	Hs.99145	KIAA library	1
Wdr1	Known	Hs.85100	Promotion of actin degeneration	1
FTL	Known	Hs.433670	Ferritin	1
SEC61B	Known	Hs.191887	Sec61 beta subunit	1
SLC25A3	Known	Hs.290404		2
KIAA0759	Unknown	Hs.7285	KIAA library	1
WIPI49	Known	Hs.9398	WD40 repeat protein interacting with PI of 49kd	1
Chromosome 16, RP11-27L11	Unknown			1
Transcribed locus	Unknown			1

### Overexpression of TGF-β1, P-gp, and membranous PKCα in pancreatic cancer tissues

**Figure 9:**
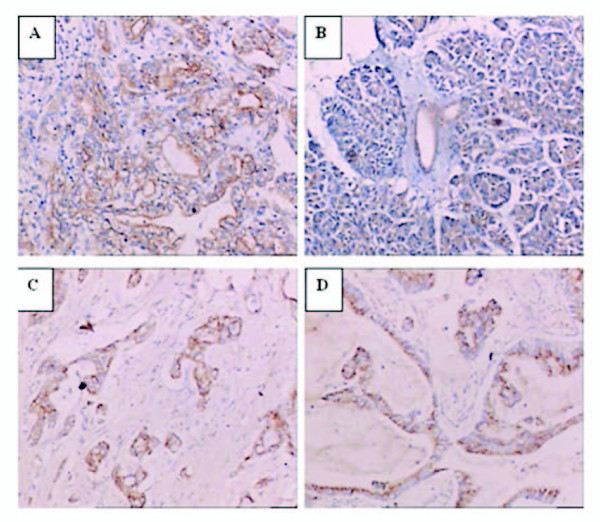
**Immunohistochemical analysis**. Representative staining of membranous PKCα (A) in pancreatic cancer tissues, cytoplasmic PKCα (B) in normal pancreas, P-gp (C) in pancreatic cancer tissues, and TGF-β1 (D) in pancreatic cancer tissues.

To determine the expression levels of TGF-β1, P-gp, and PKCα in human samples in ex vivo, we immunostained sections of pancreatic cancer tissues and the corresponding non-cancerous tissues from 42 patients. As shown in Table 2 and Figure 9, we observed overexpression of TGF-β1, P-gp, and membranous PKCα in pancreatic cancer tissues. Specifically, tumor cells showed a significantly higher rate of membranous staining for PKCα than non-neoplastic ductal cells (p < 0.01) (Table 2 and Figure 9A). In non-neoplastic ductal cells, PKCα stained weakly, and positive signals were mostly located in the cytoplasm (Figure 9B). Moreover, staining for TGF-β1 and P-gp was mainly localized in the cytoplasm of tumor cells (Figure 9C &9D). TGF-β1 was diffusely expressed in most tumor tissues (80.9%), but weakly stained in normal ductal cells (19.1%). Again, P-gp expression was found in 35 cases (83.3%) of tumor tissues, while P-gp was weakly positive in the non-neoplastic pancreas (11.9%)(Table 2).

**Table 2:** Distribution of P-gp, TGF-β1 and PKCα expression between pancreatic carcinomas and corresponding non-cancer tissues

Group	P-gp	TGF-β1	PKCα	
			Membrane	Plasma
Carcinoma	35 (83.3%)	34 (80.9%)	25 (59.5%)	22 (52.3%)
Non-cancer	7 (16.7%)*	8 (19.1%)*	2 (4.8%)*	35 (83.3%)*

*P < 0.01

We then correlated the expression data with the patients' clinicopathological findings (Table 3) and found that PKCα expression was not correlated with histological type, tumor stage or nodal status. However, we did find that the expression levels of both TGF-β1 and P-gp are associated with poor differentiation of tumors (p < 0.05). In addition, PKCα expression is correlated with expression of TGF-β1 and P-gp (RR = 0.465 and 0.412, p < 0.01, respectively), and expression of TGF-β1 with P-gp expression (RR = 0.759, p < 0.01)(Table 4, 5).

**Table 3:** Assocaition between TGF-β1, m-PKCα, or P-gp expression and clinicopathological factors

Variable	Number of patients	TGF-β1		Membranous PKCα		P-gp	
		+	%	+	%	+	%
**Differentiation**							
Well	7	5	71.4	3	42.9	6	85.7
Intermediate	30	28	93.3	20	66.7	27	96.7
Poor	5	1	20	2	40	2	40
**LN metastasis**							
Positive	13	9	69.2	7	77.8	10	76.9
Negative	39	25	86.2	18	65.5	25	93.1
**Neural invasion**							
Positive	13	9	69.2	5	77.8	11	84.6
Negative	29	25	86.2	20	80	24	82.7
**Metastatis**							
Positive	11	7	63.6	6	85.7	8	72.7
Negative	31	27	87.1	19	70.4	27	93.5

**Table 4:** Correlation between P-gp, TGF-β1 or membranous PKCα expression in pancreatic cancer

TGF-β1	Membranous PKCα		p-value	P-gp		p-value
	+	-		+	-	
+	24	10	< 0.01	33	1	< 0.01
-	1	7		2	6	
Total	25	17		35	7	

**Table 5:** Correlation between P-gp and membranous PKCα expression in pancreatic cancer

P-gp	Membranous PKCα		p-value
	+	-	
+	24	11	< 0.01
-	1	6	
Total	25	17	

## Discussion

In this study, we determined the role of TGF-β1 and its signaling pathway in regulating the growth and sensitivity to chemotherapeutic drugs of pancreatic cancer cells. We found that induction of TGF-β1 expression reduced tumor cell growth, but promoted tumor cell migration. Furthermore, pretreatment of tumor cells with TGF-β1 induced resistance to the chemotherapeutic drug cisplatin in pancreatic cancer, which was mainly mediated by PKCα and P-gp. However, inhibition of PKCα by its inhibitor Gö6976 or knockdown of TβRII by siRNA reversed the resistance of BxPC3 cells to gemcitabine, even in the presence of TGF-β1. Immunostaining showed that pancreatic cancer tissues overexpress TGF-β1 and P-gp compared to non-cancerous tissues. In addition, TGF-β1 expression was associated with P-gp and membranous PKCα expression in pancreatic cancer tissues. The data from the current study demonstrate that TGF-β1-induced drug resistance in pancreatic cancer cells was associated with PKCα expression. Our findings suggest that the PKCα inhibitor Gö6976 could be a promising sensitizer for chemotherapy in pancreatic cancer.

Overexpression of TGF-β1 in pancreatic cancer cells, either by gene transfection or by addition of recombinant TGF-β1, enhances tumor cell resistance to cisplatin. There are several potential molecular mechanisms that could be responsible for this drug resistance. For example, Warenius et al reported that upregulated cyclinD1 might be responsible for cis-diamminedichloroplatinum (CDDP) resistance in cancer cells [20], and Zhang et al suggested that the cell cycle inhibitor p21^waf1 ^might synergize with bcl-2 to confer drug resistance by inhibiting anti-cancer drug induced-apoptosis [21]. Indeed, our study shows that a reduced S phase of the cell cycle is associated with decreased cyclinD1 and increased p21^waf1 ^expression after TGF-β1 treatment. Furthermore, our data show that TGF-β1 induces expression of α-SMA, a marker of the epithelial-to-mesenchymal transition, which often results in drug resistance in cancer cells [18,19,22-24]. In addition to induction of α-SMA expression, we also found modulation of other stroma-related molecules (such as fibronectin, APLP2, and PLOD2) by TGF-β1 transfection. These data may indicate that TGF-β1-induced effects on the epithelial-to-mesenchymal transition contribute to drug resistance in pancreatic cancer.

In addition, we found that PKCα is also involved in the drug resistance of pancreatic cancer. SSH screening revealed that PKCα is upregulated by TGF-β1 via the Smad4-independent pathway. The role of PKCα in cancer drug resistance has been under investigation for decades [25,26]. Our data show that TGF-β1 induces PKCα expression in a time- and dose-dependent manner, suggesting that PKCα is indeed regulated by TGF-β1. PKCα cooperates with P-gp in drug resistance by upregulating or phosphorylating P-gp protein [27-30]. In line with the increased PKCα level, we found that P-gp expression is also elevated. Immunohistochemical data show higher levels of TGF-β1 and P-gp expression in pancreatic cancer tissues than in normal ductal cells. O'Driscoll *et al *demonstrated that pancreatic cancers expressed high levels of P-gp protein, rather than another multidrug resistance-associated protein MRP-1 [31]. In pancreatic cancer cell lines, P-gp expression was also shown elevated at different levels [32]. Our findings provide direct evidence that TGF-β1 and P-gp are functionally related.

Although we observed no remarkable difference in PKCα expression between cancerous and normal tissues of the pancreas, we did observe that membranous staining of PKCα was more obvious and was significantly correlated with P-gp expression in tumor tissues. Different subcellular localizations of PKCα cause different biological activities [33-36]. In mediating drug resistance, PKCα translocates from the cytoplasm to the membrane, phosphorylates the linker region of P-gp, activates the pump (P-gp), and subsequently causes reduction of intracellular drug accumulation. In this respect, the membrane-associated PKCα should be considered as the functional form that coordinates with P-gp. TGF-β1 inhibits the growth of PC3 (a prostate cancer cell line with wild-type Smad4) by decreasing the membrane-associated PKCα, not by altering the total level of PKCα [37]. Another study showed that TGF-β1 suppressed PTEN expression in Smad4-null pancreatic cancer cells by activating PKCα [38]. These data suggest that the existence of Smad4 may repress the Smad4-independent pathway of TGF-β1 by inhibiting functions of several modulators (such as PKCα).

Therefore, we propose that a Smad4-independent TGF-β1 pathway may promote the drug resistant phenotype in pancreatic cancer through PKCα and P-gp. Studies have shown that the MAPK and ERK pathway may be the downstream signaling pathways activated by TGF-β1. Several studies showed that p38 and ERK pathways might mediate Smad4-independent TGF-β1 responses [39-41]. Our data show that TGF-β1 treatment induces phosphorylation of p38 but not ERK1/2. We believe that in absence of Smad4 (BxPC3 cells lack of Smad4 expression) TGF-β1 activates p38 but not ERK1/2 as a transient mediator in its signaling cascades.

Indeed, we found that inhibition of PKCα or silence of TβRII reverses the resistance of BxPC3 cells to the chemotherapeutic drugs gemcitabine and cisplatin, suggesting that the PKCα inhibitor Gö6976 is a potential sensitizer to chemotherapy. Inhibition of PKCα function has been shown to effectively restore the drug-sensitive phenotype of cancer cells [42]. The PKCα inhibitor used in this study is a small molecule that has been reported to effectively abrogate DNA damage-induced cell cycle arrest and induce apoptosis [43]. In addition, we found that targeting TβRII by using siRNA did not achieve the same effect as Gö6976; it merely helped reverse gemcitabine resistance to a certain extent. However, tumor cells still remained tolerant to gemcitabine treatment. Another study demonstrated that the blockade of TβRII could not completely shut down the pathway, which may be because TβRI itself may be sufficient to transmit the TGF-β1 signal [43]. All of these findings suggest reasons why the PKCα inhibitor might be more effective in re-sensitizing cancer cells to cisplatin than that of TβRII silencing.

In summary, we have demonstrated that TGF-β1-induced drug resistance in pancreatic cancer was mediated by upregulation of both PKCα and P-gp expression and by induction of the epithelial-to-mesenchymal transition. The PKCα inhibitor Gő6976, but not TβRII silencing, restores the sensitivity of pancreatic cancer cells to cisplatin or gemcitabine. Therefore, our findings may cast new light on a future application of the PKCα inhibitor as a sensitizer in chemotherapy for pancreatic cancer.

## List of abbreviations

TGF-β1: Transforming Growth Factor-beta 1; PKCα: Protein Kinase C-alpha; TβRII: Transforming Growth Factor-beta 1 receptor II; SSH: Suppressive Subtracted Hybridization; P-gp: P-glycoprotein; MDR1: Multidrug Resistant Gene 1; ABC: ATP-binding cassette; DTH: Dihydrotestosterone; ECM: Extracellular Matrix; EMT: Epithelial-to-Mesenchymal Transition; CDDP: Cis-diamminedichloroplatinum

## Competing interests

The authors declare that they have no competing interests.

## Authors' contributions

YC carried out the molecular genetic studies, participated in the sequence alignment and drafted the manuscript. GY participated in the design of the study and performed the statistical analysis. DY carried out the immunoassay and participated in the sequence alignment. MZ conceived of the study, and participated in its design and coordination and helped to draft the manuscript. All authors read and approved the final manuscript.
